# The Accuracy of Single MicroRNAs in Peripheral Blood to Diagnose Ovarian Cancer: An Updated Meta-Analysis

**DOI:** 10.1155/2020/1075942

**Published:** 2020-01-13

**Authors:** Yubao Cui, Shanchao Hong, Xuming Zhu

**Affiliations:** Department of Clinical Laboratory, Wuxi People's Hospital Affiliated to Nanjing Medical University, Wuxi, Jiangsu Province, China

## Abstract

**Background:**

Ovarian cancer is the 5th leading cause of death of women due to cancer in the United States. Although carbohydrate antigen 125 has a moderate diagnostic utility, the phenomenon of false-positive exists. As novel effective biomarkers, some single microRNAs (miRNAs) have diagnostic values for ovarian cancer, but the results lack consistency. In order to precisely and comprehensively assess the diagnostic value of single miRNAs for ovarian cancer, a meta-analysis is performed.

**Methods:**

Articles concerning the diagnostic value of single miRNAs for ovarian cancer were searched from databases. The pooled sensitivity (SEN), specificity (SPE), positive likelihood ratio (PLR), negative likelihood ratio (NLR), and diagnostic odds ratio (DOR) with the corresponding 95% confidence interval (CI) were calculated. Area under curve (AUC) of the summary receiver-operating characteristic (SROC) curve was also calculated.

**Results:**

In total, 22 studies including 8 kinds of single miRNAs were enrolled in this paper (6 studies for miR-200c, 3 studies for miR-200a and miR-200b, and 2 studies for miR-205, miR-145, miR-141, miR-429, and miR-125b). For miR-200c, the pooled SEN and SPE were, respectively, 0.768 (95% CI: 0.722-0.811) and 0.680 (95% CI: 0.624-0.732); the pooled PLR and NLR were, respectively, 2.897 (95% CI: 1.787-4.698) and 0.340 (95% CI: 0.276-0.417); the pooled DOR was 8.917 (95% CI: 4.521-17.587); and AUC of SROC curve was 0.815. For miR-200a, the pooled SEN and SPE were, respectively, 0.759 (95% CI: 0.670-0.833) and 0.717 (95% CI: 0.627-0.795); the pooled PLR and NLR were, respectively, 3.129 (95% CI: 0.997-9.816) and 0.301 (95% CI: 0.207-0.437); the pooled DOR was 11.323 (95% CI: 3.493-36.711); and AUC of SROC curve was 0.857. For miR-200b, the pooled SEN and SPE were, respectively, 0.853 (95% CI: 0.776-0.912) and 0.775 (95% CI: 0.690-0.846); the pooled PLR and NLR were, respectively, 4.327 (95% CI: 0.683-27.415) and 0.225 (95% CI: 0.081-0.625); the pooled DOR was 19.678 (95% CI: 2.812-137.72); and AUC of SROC curve was 0.90. For miR-205, miR-145, miR-141, miR-429, and miR-125b, each diagnostic value should be interpreted cautiously because only two studies were included.

**Conclusions:**

miR-200c, miR-200a, and miR-200b can be useful diagnostic biomarkers for ovarian cancer. More related studies are needed for miR-205, miR-145, miR-141, miR-429, and miR-125b.

## 1. Introduction

Ovarian cancer is the 5th leading cause of death of women due to cancer in the United States [1]. Ovarian cancer is often at an advanced stage by the time of diagnosis and has metastasized throughout the peritoneal cavity [2]. The overall 5-year survival rate of this fatal disease is only 45.6% [3]. A widely used biomarker to diagnose ovarian cancer is carbohydrate antigen 125 (CA125). With the sensitivity and specificity of, respectively, 0.78 and 0.84 [4], CA125 has a moderate diagnostic utility. However, elevated serum CA125 levels may be observed in other physiological or pathological conditions (menstruation, pregnancy, endometriosis, and inflammatory diseases of the peritoneum) [5], resulting in false-positive. Therefore, it is necessary to find novel effective biomarkers to diagnose ovarian cancer.

MicroRNAs (miRNAs), a family of small noncoding RNAs (19 ± 22 nucleotides), have shown great promise as biomarkers for cancer due to their stability and relative easiness to accurately measure [6]. Recently, some articles have reported that some single miRNAs have diagnostic values for ovarian cancer [7–17], but the results lack consistency. Take miR-429 as an example, Márton et al. demonstrated that the sensitivity and specificity to diagnose ovarian cancer were, respectively, 0.857 and 0.683 [9], while Meng et al. found that the sensitivity and specificity were, respectively, 0.594 and 0.955 [14]. In order to precisely and comprehensively assess the diagnostic value of single miRNAs for ovarian cancer, this meta-analysis is performed.

## 2. Material and Methods

This meta-analysis was conducted according to the guidelines of the PRISMA statement ([Supplementary-material supplementary-material-1]) [18].

### 2.1. Search Strategy

We systematically searched articles published prior to September 23, 2019 in the databases of PubMed, Embase, and Web of Knowledge. Search terms were as follows: (“microRNA” or “miRNA” or “miR”) and (“ovarian cancer” or “ovarian carcinoma” or “ovarian neoplasm” or “ovarian cancers” or “ovarian carcinomas” or “ovarian neoplasms” or “cancer of ovary” or “carcinoma of ovary” or “neoplasm of ovary” or “cancers of ovary” or “carcinomas of ovary” or “neoplasms of ovary”) and (“diagnosis” or “sensitivity” or “specificity” or “diagnosis” or “plasma” or “serum” or “blood” or “circulating”). After relevant articles were identified, we examined their cited references to select other relevant articles. Two reviewers independently searched the articles. Any disagreements between the two reviewers were resolved by a third author.

### 2.2. Selection Criteria

Inclusion criteria were as follows: (1) articles were designed to evaluate the accuracy of single miRNAs for diagnosing ovarian cancer; (2) 2 × 2 contingency tables could be directly extracted or calculated from the articles; (3) at least two studies concerning the same single miRNA; and (4) articles were written in English.

Exclusion criteria were as follows: (1) reviews, meta-analyses, letters, or expert opinions; (2) combined panels of different single miRNAs; and (3) overlapped publication article.

### 2.3. Data Extraction

The following data were extracted: first author, year of publication, country of participants, source of control group, sample size, single miRNAs as biomarker, miRNAs as control for normalization, relative expression in the case group compared to the control group, diagnostic sensitivity (SEN) and specificity (SPE) values, and the values of true positive (TP), false positive (FP), false negative (FN), and true negative (TN).

### 2.4. Quality Assessment

To assess the quality of each included study, we used the quality assessment of diagnostic accuracy studies-2 (QUADAS-2) tool [19]. QUADAS-2 comprises four key domains, including patient selection, index test, reference standard, and the timing of the index and reference tests (flow and timing). These four domains were used to evaluate the risk of bias, and the first three domains were applied to assess applicability concerns. Two reviewers independently assessed the quality. Any disagreements between the two reviewers were resolved by a third author.

### 2.5. Statistical Analysis

The pooled sensitivity (SEN), specificity (SPE), positive likelihood ratio (PLR), negative likelihood ratio (NLR), diagnostic odds ratio (DOR), and their corresponding 95% confidence interval (CI) were calculated. Area under curve (AUC) of the summary receiver-operating characteristic curve (SROC) was also calculated. A Cochrane-*Q* test of heterogeneity was performed using the inconsistency index. The value of *I*^2^ > 50% and *P* value < 0.05 indicated the existence of significant heterogeneity among studies. If heterogeneity was detected, the random effects model was used; otherwise, the fixed effects model was used. Deeks' funnel plot asymmetry analysis was performed to identify publication bias. Sensitivity analysis was conducted to assess the stability of our analysis. Statistical analyses were undertaken using Stata software version 12.0 (College Station, TX, USA), Meta-DiSc XI for Windows (Cochrane Colloquium, Barcelona, Spain), and RevMan 5.3 (Cochrane Collaboration, Oxford, UK).

## 3. Results

### 3.1. Literature Search and Study Characteristics

**Figure 1:**
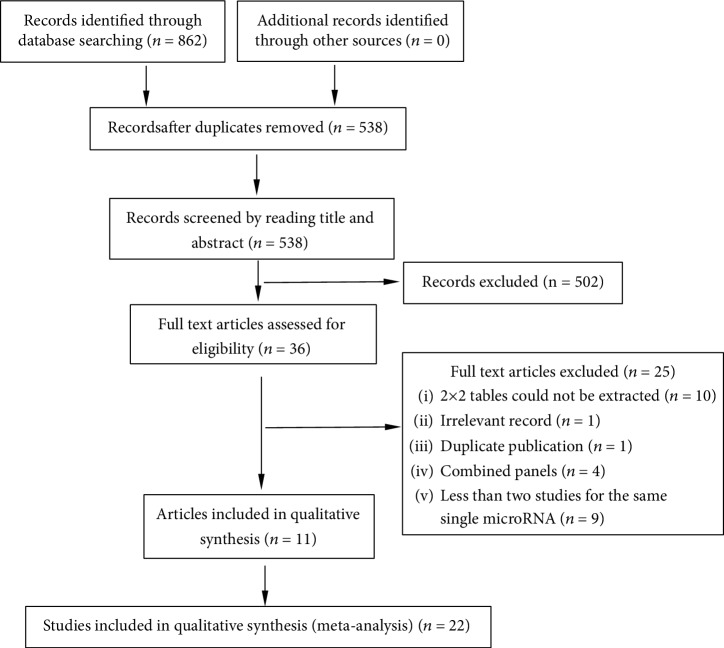
Flow diagram of study selection.

A total of 862 articles were identified through database searching. After removing duplicates, the titles and abstracts for 538 records were screened for eligibility. Of these, 36 records were identified and full-text articles were retrieved. 25 manuscripts were then excluded through assessment of the full-text articles, and 11 remaining articles encompassing 22 studies were included in the meta-analysis (Figure 1).

These 22 studies included 2667 participants (1485 ovarian cancer patients and 1182 controls). Their characteristics and the numbers of TP, FP, FN, and TN are listed in Table 1. There were 8 kinds of single miRNAs enrolled in this paper (miR-200a, miR-200b, miR-200c, miR-205, miR-145, miR-141, miR-429, and miR-125b). The study number of miR-200c was six, the number of miR-200a and miR-200b were both two, and the number of miR-205, miR-145, miR-141, miR-429, and miR-125b were all two. Countries of participants included China, Korea, Hungary, India, Germany, and Australia. The specimen of all studies came from serum (including serum exosome) or plasma. Three studies regarded patients with benign disease as control groups, and the other 18 studies regarded healthy people as control groups. Except for one study, relative expressions of miRNAs in the case group were higher than those in the control group.

### 3.2. Methodological Quality Assessment

**Figure 2:**
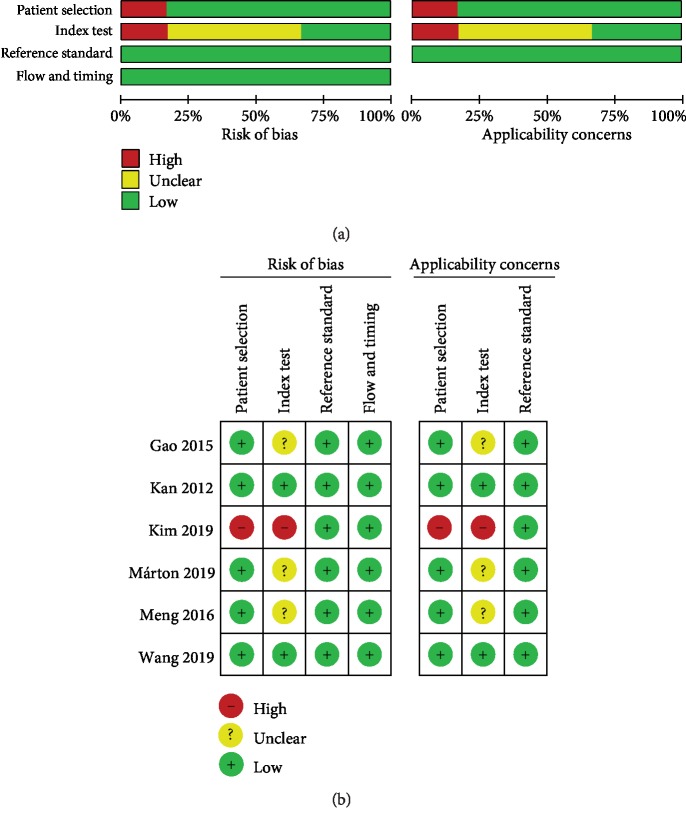
Quality assessment of miR-200c according to QUADAS-2 guidelines: (a) graph and (b) summary.

Quality assessment of included studies was conducted using the QUADAS-2 tool. For miR-200c (Figure 2), high risk and unclear situation existed in patient selection and index test. For miR-200a, miR-200b, miR-205, miR-145, miR-141, miR-429, and miR-125b (Figures [Supplementary-material supplementary-material-1]–[Supplementary-material supplementary-material-1]), the majority of included studies satisfied most domains of QUADAS-2. The high risk and unclear situation existed in the domains of patient selection and index test due to variances in the control group, specimen, and miRNAs as control for normalization.

### 3.3. Diagnostic Values

**Figure 3:**
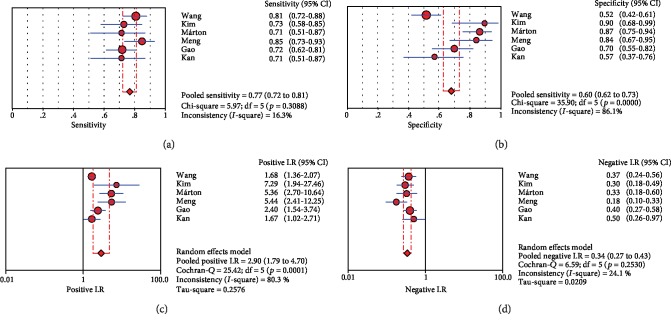
Forest plots of miR-200c: (a) sensitivity, (b) specificity, (c) positive likelihood ratio, and (d) negative likelihood ratio.

For miR-200c, the pooled SEN and SPE were, respectively, 0.768 (95% CI: 0.722-0.811) and 0.680 (95% CI: 0.624-0.732) (Figure 3). *I*^2^ for SEN was 16.3% (*P* = 0.308), showing no significant heterogeneity among studies. The pooled PLR and NLR were, respectively, 2.897 (95% CI: 1.787-4.698) and 0.340 (95% CI: 0.276-0.417) (Figure 3). The pooled DOR was 8.917 (95% CI: 4.521-17.587). AUC of SROC curve was 0.815.

For miR-200a (Table 2), the pooled SEN and SPE were, respectively, 0.759 (95% CI: 0.670-0.833) and 0.717 (95% CI: 0.627 - 0.795). *I*^2^ for SEN was 66.1% (*P* = 0.052), showing significant heterogeneity among studies. The pooled PLR and NLR were, respectively, 3.129 (95% CI: 0.997-9.816) and 0.301 (95% CI: 0.207-0.437). The pooled DOR was 11.323 (95% CI: 3.493-36.711). AUC of SROC curve was 0.857.

For miR-200b (Table 2), the pooled SEN and SPE were, respectively, 0.853 (95% CI: 0.776-0.912) and 0.775 (95% CI: 0.690-0.846). *I*^2^ for SEN was 78.1% (*P* = 0.01), showing significant heterogeneity among studies. The pooled PLR and NLR were, respectively, 4.327 (95% CI: 0.683-27.415) and 0.225 (95% CI: 0.081-0.625). The pooled DOR was 19.678 (95% CI: 2.812-137.72). AUC of SROC curve was 0.90.

Among miR-205, miR-145, miR-141, miR-429, and miR-125b, miR-145 had the highest diagnostic value (pooled SEN and SPE of, respectively, 0.958 and 0.933; pooled PLR and NLR of, respectively, 11.711 and 0.043; and pooled DOR of 278.62). For these 5 miRNAs, the AUC of SROC curve could not be obtained due to only two studies included (Table 2).

### 3.4. Publication Bias

**Figure 4:**
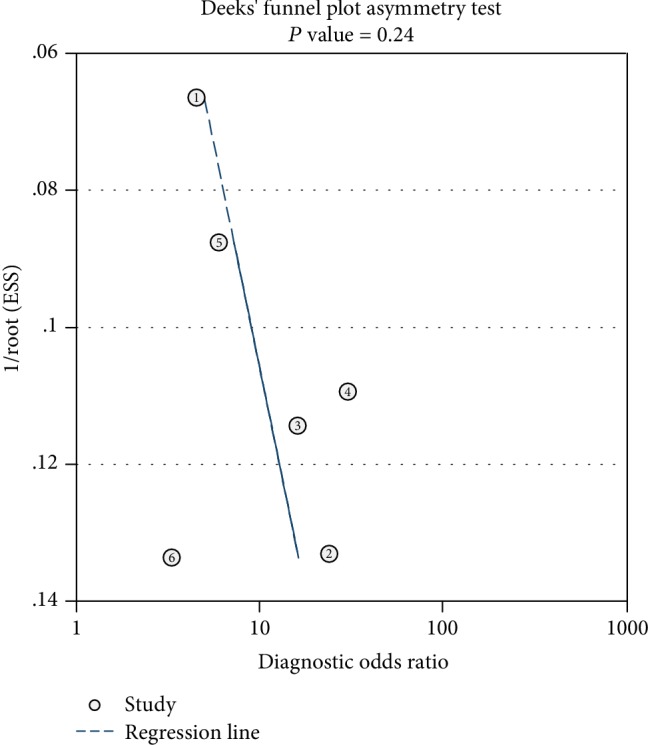
Deeks' funnel plot for miR-200c.

As shown in Deeks' funnel plot (Figure 4), the plot of miR-200c had a symmetrical funnel shape, revealing that publication bias was not absent. Furthermore, the *P* value for Deeks' funnel plot asymmetry test was 0.24, indicating a lack of publication bias in this meta-analysis.

For miR-200a, miR-200b, miR-205, miR-145, miR-141, miR-429, and miR-125b, publication bias could not be assessed due to the small number of studies included.

### 3.5. Sensitivity Analysis

Sensitivity analysis was performed by eliminating studies one by one ([Supplementary-material supplementary-material-1]). For miR-200c, the pooled SEN and corresponding heterogeneities were not dominant and a study by Márton et al. [9] may be the source of heterogeneities. For miR-200a, miR-200b, miR-205, miR-145, miR-141, miR-429, and miR-125b, sensitivity analysis could not be performed due to the small number of studies included.

## 4. Discussion

Mir-200c, a member of the miR-200 family, is an illustrious tumor suppressor and one of the highly studied miRNAs in terms of cancer development, proliferation, therapy resistance, and metastasis [20]. In our paper, the pooled SEN and SPE of miR-200c were, respectively, 0.768 and 0.680, presenting a similar diagnostic ability with CA125. The pooled PRL of 2.897 indicated that the probability to be ovarian cancer was 2.897-fold increased with a positive miRNA result. Conversely, the pooled NRL of 0.34 showed that the probability could decrease 66% when the miRNA result was negative. With the DOR value of 8.917 and an AUC of SROC of 0.815, miR-200c could be a useful biomarker to diagnose ovarian cancer. Mir-200c was included in more studies, and the diagnostic values of Mir-200c were more convincing than other miRNAs in our paper.

miR-200a is also a member of the mir-200 family, one kind of long noncoding RNA that can promote invasion and metastasis of ovarian cancer through miR-200a [21]. Our paper enrolled 3 studies concerning miR-200a and showed that the diagnostic value of miR-200a was better than miR-200c.

As another member of the mir-200 family, miR-200b may improve the chemotherapeutic efficacy of ovarian cancer by targeting DNA methyl transferases [22]. Our paper enrolled 3 studies concerning miR-200b and showed that the diagnostic value of miR-200b was better than miR-200a and miR-200c.

Among miR-205, miR-145, miR-141, miR-429, and miR-125b, miR-145 had the highest diagnostic value. Because only 2 studies were included for these 5 miRNAs, the diagnostic values should be interpreted cautiously.

We noticed that three meta-analyses have also reported the diagnostic value of miRNAs for ovarian cancer [23–25]. We read them with great interest. For the meta-analysis by Wu et al. [23], it enrolled 11 studies concerning 5 single miRNAs (2 studies for miR-200a, 2 studies for miR-200b, 3 studies for miR-200c, 2 studies for miR-429, and 2 studies for miR-25) and combined them together to diagnose ovarian cancer. It is unreasonable to take all single miRNAs as a diagnostic biomarker for ovarian cancer because results of different single miRNAs cannot be combined in a meta-analysis. For the other two meta-analyses [24, 25], they also combined different single miRNAs to diagnose ovarian cancer. Compared to these previous meta-analyses, our paper only assessed the diagnostic value of the same single miRNA from different studies, which is more reasonable and practical.

There were several limitations in our meta-analysis. First, a high risk and an unclear situation existed in patient selection and an index test could lower methodological qualities. Second, significant heterogeneities existed among included studies. Third, our paper only enrolled studies concerning serum-based and plasma-based specimens but not other specimens such as tissue-based or urine-based specimens. Fourth, there was a small number of included studies for some single miRNAs. It is necessary to adopt a standardization of the control group and specimens and to conduct more related studies in the future.

Our meta-analysis showed that for ovarian cancer, miR-200c, miR-200a, and miR-200b can be useful diagnostic biomarkers. For miR-205, miR-145, miR-141, miR-429, and miR-125b, their value as diagnostic biomarkers should be interpreted cautiously.

## Figures and Tables

**Table 1:** Characteristics of included studies.

Author	Year	Country	Specimen	Control group	miRNA as biomarker	Case size	Control size	miRNA as control	Relative expression^∗^	SEN	SPE	TP	FP	FN	TN
Wang et al. [7]	2019	China	Serum	HP	miR-200c	110	116	miR-103	High	0.809	0.517	89	56	21	60
Wang et al. [7]	2019	China	Serum	HP	miR-205	110	116	miR-103	High	0.764	0.526	84	55	26	61
Kim et al. [8]	2019	Korea	Serum exosome	PBOD	miR-200c	48	20	RNU48	High	0.729	0.9	35	2	13	18
Kim et al. [8]	2019	Korea	Serum exosome	PBOD	miR-145	48	20	RNU48	High	0.917	0.868	44	3	4	17
Márton et al. [9]	2019	Hungary	Plasma	HP	miR-200a	28	60	miR-103	High	0.857	0.783	24	13	4	47
Márton et al. [9]	2019	Hungary	Plasma	HP	miR-200b	28	60	miR-103	High	0.679	0.9	19	6	9	54
Márton et al. [9]	2019	Hungary	Plasma	HP	miR-200c	28	60	miR-103	High	0.714	0.867	20	8	8	52
Márton et al. [9]	2019	Hungary	Plasma	HP	miR-141	28	60	miR-103	High	0.857	0.9	24	6	4	54
Márton et al. [9]	2019	Hungary	Plasma	HP	miR-429	28	60	miR-103	High	0.857	0.683	24	19	4	41
Zuberi et al. [10]	2019	India	Serum	HP	miR-145	70	70	RNU6B	Low	0.991	0.957	69	3	1	67
Meng et al. [11]	2016	Germany	Serum	HP	miR-200a	60	32	miR-484	High	0.672	0.9	40	3	20	29
Meng et al. [11]	2016	Germany	Serum	HP	miR-200b	60	32	miR-484	High	0.931	0.9	56	3	4	29
Meng et al. [11]	2016	Germany	Serum	HP	miR-200c	60	32	miR-484	High	0.845	0.85	51	5	9	27
Zhu et al. [12]	2017	China	Serum	PBOD	miR-125b	135	54	miR-16	High	0.756	0.685	102	17	33	37
Zuberi et al. [13]	2016	India	Serum	HP	miR-125b	70	70	RNU6	High	0.623	0.771	44	16	26	54
Meng et al. [14]	2015	Germany	Serum	HP	miR-429	180	66	miR-484	High	0.594	0.955	107	3	73	63
Gao and Wu [15]	2015	China	Serum	HP	miR-141	93	50		High	0.69	0.72	64	14	29	36
Gao and Wu [15]	2015	China	Serum	HP	miR-200c	93	50		High	0.72	0.7	67	15	26	35
Zheng et al. [16]	2013	China	Plasma	HP	miR-205	134	70		High	0.301	0.942	40	4	94	66
Kan et al. [17]	2012	Australia	Serum	HP	miR-200a	28	28	miR-103	High	0.857	0.357	24	18	4	10
Kan et al. [17]	2012	Australia	Serum	HP	miR-200b	28	28	miR-103	High	0.857	0.357	24	18	4	10
Kan et al. [17]	2012	Australia	Serum	HP	miR-200c	28	28	miR-103	High	0.714	0.571	20	12	8	16

Abbreviation: HP—healthy people; PBOD—patients with benign ovarian disease; SEN—sensitivity; SPE—specificity; TP—true positive; FP—false positive; FN—false negative; TN—true negative. ^∗^Relative expression for microRNA as a biomarker of the case group compared to the control group.

**Table 2:** Diagnostic values of miRNAs for ovarian cancer.

MicroRNA	*n*	SEN (95% CI)	SPE (95% CI)	PLR (95% CI)	NLR (95% CI)	DOR (95% CI)	AUC
miR-200a	3	0.759 (0.670-0.833)	0.717 (0.627-0.795)	3.129 (0.997-9.816)	0.301 (0.207-0.437)	11.323 (3.493-36.711)	0.857
miR-200b	3	0.853 (0.776-0.912)	0.775 (0.690-0.846)	4.327 (0.683-27.415)	0.225 (0.081-0.625)	19.678 (2.812-137.72)	0.90
miR-200c	6	0.768 (0.722-0.811)	0.680 (0.624-0.732)	2.897 (1.787-4.698)	0.340 (0.276-0.417)	8.917 (4.521-17.587)	0.815
miR-205	2	0.508 (0.444-0.573)	0.683 (0.611-0.749)	2.675 (0.740-9.672)	0.590 (0.310-1.122)	4.359 (2.716-6.996)	
miR-145	2	0.958 (0.904-0.986)	0.933 (0.861-0.975)	11.711 (3.108-44.130)	0.043 (0.004-0.461)	278.62 (11.704-6632.9)	
miR-141	2	0.727 (0.639-0.804)	0818 (0.733-0.885)	4.411 (1.298-14.988)	0.281 (0.093-0.856)	16.279 (1.797-147.47)	
miR-429	2	0.630 (0.560-0.696)	0.825 (0.748-0.887)	5.700 (0.618-52.568)	0.331 (0.148-0.736)	21.999 (9.595-50.438)	
miR-125b	2	0.712 (0.645-0.773)	0.734 (0.647-0.809)	2.539 (1.870-3.447)	0.420 (0.331-0.533)	6.219 (3.819-10.127)	

Abbreviation: SEN—sensitivity; SPE—specificity; PLR—positive likelihood ratio; NLR—negative likelihood ratio; DOR—diagnostic odds ratio; AUC—area under curve; CI—confidence interval.

## Data Availability

The [DATA TYPE] data supporting this [SYSTEMATIC REVIEW or META-ANALYSIS] are from previously reported studies and datasets, which have been cited. The processed data are available [AT REPOSITORY NAME/IN THE SUPPLEMENTARY FILES/FROM THE CORRESPONDING AUTHOR UPON REQUEST].

## Supplementary Materials

Supplementary Materials Table S1: PRISMA checklist. Table S2: sensitivity analysis for pooled sensitivities and corresponding heterogeneities. Figure S1: quality assessment of miR-200a according to QUADAS-2 guidelines. (A) Graph. (B) Summary. Figure S2: quality assessment of miR-200b according to QUADAS-2 guidelines. (A) Graph. (B) Summary. Figure S3: quality assessment of mir-205 according to QUADAS-2 guidelines. (A) Graph. (B) Summary. Figure S4: quality assessment of mir-145 according to QUADAS-2 guidelines. (A) Graph. (B) Summary. Figure S5: quality assessment of mir-141 according to QUADAS-2 guidelines. (A) Graph. (B) Summary. Figure S6: quality assessment of mir-429 according to QUADAS-2 guidelines. (A) Graph. (B) Summary. Figure S7: quality assessment of mir-125b according to QUADAS-2 guidelines. (A) Graph. (B) Summary.
